# Correlation research of susceptibility single nucleotide polymorphisms and the severity of clinical symptoms in attention deficit hyperactivity disorder

**DOI:** 10.3389/fpsyt.2022.1003542

**Published:** 2022-09-23

**Authors:** Yunyu Xu, Shuangxiang Lin, Jiejie Tao, Xinmiao Liu, Ronghui Zhou, Shuangli Chen, Punit Vyas, Chuang Yang, Bicheng Chen, Andan Qian, Meihao Wang

**Affiliations:** ^1^Department of Radiology, The First Affiliated Hospital of Wenzhou Medical University, Wenzhou, China; ^2^Department of Radiology, The Second Affiliated Hospital Zhejiang University School of Medicine, Hangzhou, China; ^3^School of Laboratory Medicine and Life Sciences, Wenzhou Medical University, Wenzhou, China; ^4^School of Medicine, Indiana University, Indianapolis, IN, United States; ^5^Department of Psychiatry, The First Affiliated Hospital of Wenzhou Medical University, Wenzhou, China; ^6^Key Laboratory of Diagnosis and Treatment of Severe Hepato-Pancreatic Diseases of Zhejiang Province, Zhejiang Provincial Top Key Discipline in Surgery, The First Affiliated Hospital of Wenzhou Medical University, Wenzhou, China

**Keywords:** attention deficit hyperactivity disorder (ADHD), single nucleotide polymorphism (SNP), GWAS, TWAS, panel, severity of clinical symptoms

## Abstract

**Objective:**

To analyze the correlation between susceptibility single nucleotide polymorphisms (SNPs) and the severity of clinical symptoms in children with attention deficit hyperactivity disorder (ADHD), so as to supplement the clinical significance of gene polymorphism and increase our understanding of the association between genetic mutations and ADHD phenotypes.

**Methods:**

193 children with ADHD were included in our study from February 2017 to February 2020 in the Children’s ADHD Clinic of the author’s medical institution. 23 ADHD susceptibility SNPs were selected based on the literature, and multiple polymerase chain reaction (PCR) targeted capture sequencing technology was used for gene analysis. A series of ADHD-related questionnaires were used to reflect the severity of the disease, and the correlation between the SNPs of specific sites and the severity of clinical symptoms was evaluated. R software was used to search for independent risk factors by multivariate logistic regression and the “corplot” package was used for correlation analysis.

**Results:**

Among the 23 SNP loci of ADHD children, no mutation was detected in 6 loci, and 2 loci did not conform to Hardy-Weinberg equilibrium. Of the remaining 15 loci, there were 9 SNPs, rs2652511 (SLC6A3 locus), rs1410739 (OBI1-AS1 locus), rs3768046 (TIE1 locus), rs223508 (MANBA locus), rs2906457 (ST3GAL3 locus), rs4916723 (LINC00461 locus), rs9677504 (SPAG16 locus), rs1427829 (intron) and rs11210892 (intron), correlated with the severity of clinical symptoms of ADHD. Specifically, rs1410739 (OBI1-AS1 locus) was found to simultaneously affect conduct problems, control ability and abstract thinking ability of children with ADHD.

**Conclusion:**

There were 9 SNPs significantly correlated with the severity of clinical symptoms in children with ADHD, and the rs1410739 (OBI1-AS1 locus) may provide a new direction for ADHD research. Our study builds on previous susceptibility research and further investigates the impact of a single SNP on the severity of clinical symptoms of ADHD. This can help improve the diagnosis, prognosis and treatment of ADHD.

## Introduction

Attention deficit hyperactivity disorder (ADHD) is a common neurodevelopmental disorder that affects between 2 and 7% of children worldwide (1, 2), and a growing body of literature supports the notion that the disease persists into adulthood in most cases (3). It seriously impairs the individual’s ability to function in academic, career and social environments, and has adverse effects on individuals, families and society as a whole (4, 5). Clinically, ADHD is characterized by a considerable degree of hyperactivity, impulsivity and inattention (6). Previous studies have shown that ADHD is usually persistent and significantly impairs health, with an increased risk of poor overall outcomes (7). Adolescents with ADHD are at risk for other psychiatric disorders in childhood, adolescence, and adulthood, including mood, anxiety, and substance use disorders, and may even lead to criminal behavior (6). Therefore, studies on the etiology and pathophysiological mechanism of ADHD have been of great scientific concern.

Some epidemiological and clinical studies have demonstrated that genetic and environmental risk factors influence the structural and functional ability of brain networks involved in behavior and cognition in the etiology of ADHD (7). Studies of families, twins and foster children suggest that ADHD is familial, in which up to 80% of the different phenotypes can be explained by genetic variation (8). In addition, there is considerable genetic overlap between ADHD and other psychiatric disorders, such as antisocial personality disorder/behavior, cognitive disorders, autism spectrum disorders, schizophrenia, bipolar disorder, and major depressive disorder (9–15). A recent cross-trait meta-analysis identified pleiotropic genomic loci responsible for ADHD, autism spectrum disorder, and intelligence. The research also found that ADHD was associated with inheriting a reduced set of low-intelligence alleles (16). Thus, genetic factors can be seen to play an important role in the development of the disease, and both common and rare genetic variants are associated with the risk of ADHD (17, 18).

Previous studies have shown that the risk of a common genetic variant of ADHD, also known as single nucleotide polymorphism (SNP) heritability, is also associated with depression, behavioral problems, schizophrenia, persistent measures of ADHD symptoms and other neurodevelopmental disorders (7). Recently, the SNP panel has been widely used in population genetics studies (19, 20). Here, based on previous susceptibility studies, we plan to further analyze the effect of SNPs on the clinical severity of ADHD.

Recently, a combined sample of 55,374 individuals from an international collaboration for genome-wide association study (GWAS) was used to identify the first genome-wide salient loci for ADHD. This genome-wide meta-analysis provided the results of 12 ADHD-associated loci and their genome-wide significant index variations (7). 11 of these loci were selected for this study based on the form of the SNPs (rs11420276, rs1222063, rs9677504, rs4858241, rs28411770, rs4916723, rs74760947, rs11591402, rs1427829, rs281324, and rs212178). On this basis, in order to strengthen the clinical diagnosis of ADHD in the case of extreme expression of one or more heritable quantitative traits, 12 other previously reported ADHD susceptibility SNPs were included according to the literature (rs3768046, rs1199039, rs11210892, rs12741964, rs2906457, rs1410739, rs223508, rs429699, rs27048, rs2652511, rs11564750, and rs10044618) (21–24). Since ADHD susceptibility SNPs were focused on, the whole gene detection was not conducted, but susceptibility loci were chosen based on previous findings: ADHD-related loci were selected via GWAS and transcriptome-wide association study (TWAS) (7, 21); at the same time, because dopamine-related genes have been considered as candidate genes for ADHD heritability (25, 26), ADHD susceptibility SNPs were also screened and selected at dopamine-related loci (22–24). For example, rs27048 and rs429699 have been reported to be genetic markers of ADHD-inattention subtype (ADHD-I) (23), and rs2652511 was found to be significantly related to ADHD-combined subtype (ADHD-C) (24). Then the amplification and extension products of twenty-three SNP sites were designed and made into a panel to detect the mutation of gene loci, so as to evaluate the influence of SNPs on the severity of clinical symptoms of ADHD.

## Materials and methods

**FIGURE 1:**
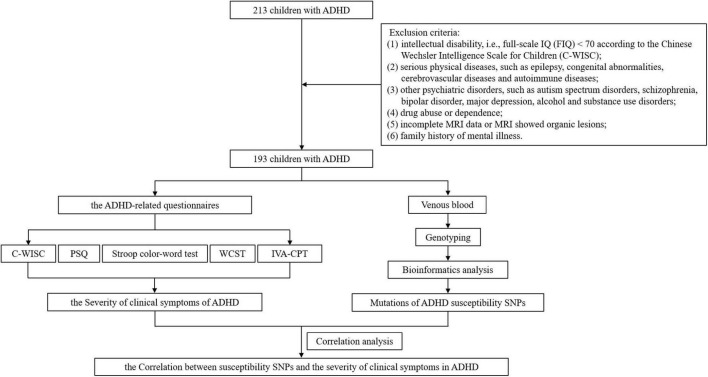
The workflow of the study.

After enrollment and exclusion criteria, different ADHD-related questionnaires, such as Conners parent symptom questionnaire, Stroop color-word test, Wisconsin card sorting test, et al., were used for assessment. The indicators from the questionnaires were then used to reflect the severity of different clinical symptoms of ADHD. DNA was extracted from venous blood for genotyping and bioinformatics analysis to obtain mutations in SNPs. Then the influence of SNPs on the severity of clinical symptoms of ADHD can be evaluated by correlational analysis between the indicators in the questionnaire results and the mutation status of SNPs. The workflow is shown in Figure 1.

### Participants

A total of 193 children with ADHD were included in our study from February 2017 to February 2020 in the Children’s ADHD Clinic of the author’s medical institution, including 163 males and 32 females, aged 6–15 (8.87 ± 2.17) years. Inclusion criteria: (1) meet the diagnostic criteria for ADHD according to the Diagnostic and Statistical Manual of Mental Disorders – the Fifth Edition (DSM-V); (2) complete questionnaire data; (3) complete magnetic resonance imaging (MRI) data, including sequences of T1-weighted imaging (T1WI), T2-weighted imaging and diffusion-weighted imaging (DWI), which showed no organic lesions. Exclusion criteria: (1) intellectual disability, i.e., full-scale IQ (FIQ) < 70 according to the Chinese Wechsler Intelligence Scale for Children (C-WISC); (2) serious physical diseases, such as epilepsy, congenital abnormalities, cerebrovascular diseases and autoimmune diseases; (3) other psychiatric disorders, such as autism spectrum disorders, schizophrenia, bipolar disorder, major depression, alcohol and substance use disorders; (4) drug abuse or dependence; (5) incomplete MRI data or MRI showed organic lesions; (6) family history of mental illness. The study, including all objectives and experimental procedures, has been approved by the Ethics Committee of Clinical Research in the First Affiliated Hospital of Wenzhou Medical University (Ethics Audit Number: KY2018-162).

### Clinical data collection

Clinical data were collected through electronic medical records. Gender, age, date of birth, grade of schooling, mode of delivery, past medical history and family history of mental illness were recorded.

### Variables and data sources

#### Chinese Wechsler intelligence scale for children

The C-WISC (27) was used to evaluate the IQ of children. The measurement results of the scale were expressed by verbal IQ (VIQ), performance IQ (PIQ) and full-scale IQ (FIQ), which were used to illustrate children’s cognitive ability. Higher IQ scores reflect better overall cognitive ability. According to their FIQ score, they were stratified into six grades: “A” means FIQ ≥ 130, “B” means 120 ≤ FIQ < 130, “C” means 110 ≤ FIQ < 120, “D” means 90 ≤ FIQ < 110, “E” means 80 ≤ FIQ < 90, “F” means 70 ≤ FIQ < 80; they would be listed by “FIQ (Grade)” in the results.

#### Conners parent symptom questionnaire

The Conners parent symptom questionnaire (PSQ) was used to assess the symptoms in children with ADHD. The scale consisted of 48 items and 6 subscales, which reflect 6 clinical phenotypes of ADHD, respectively, including conduct problems, psychosomatic disorders, anxiety, learning problems, hyperactivity/impulsivity and hyperactivity indices. Each score is based on a scale of four from 0 to 3: “0” means no exception; “1” means occasionally a little or slightly; “2” means frequent or more severe; “3” means very common or very serious. A *Z*-score was calculated by adding up the scores and dividing it by the number of items. Each phenotype was graded based on gender and PSQ score: “Y” indicates the presence of the phenotype, and “N” indicates the opposite (28, 29).

#### Stroop color-word test

The Stroop color-word test was used to assess control capabilities. The test mainly measures the ability of perceptual switching, selective attention and the ability to inhibit habitual response patterns, and is sensitive to the plasticity of mental control and response in executive function (30, 31). In the Stroop color-word test, 126 words were randomly arranged in 14 × 9 (rows × columns). The test consisted of three parts: part A named the color blocks (red, blue, green, or yellow); part B involved reading words of color printed in black (“red,” “blue,” “green,” and “yellow”); part C asked for naming the color of the printed word, which was inconsistent with the word itself (for example, the word “red” was printed in blue). There was a 60-s break after each part, and a 100 ms-long “+” was presented before the next part began. The participants were asked to respond accurately, and as quickly as possible by pressing the appropriate button. Stimuli were presented one by one. Reaction time (RT) and errors were recorded. The Stroop intervention score (IG) was obtained by formula C − [(A * B)/(A + B)]. The higher the IG, the more severe the cognitive control deficit. If the participants felt tired during the task, the rest time was extended. All participants completed the test (32–34).

#### Wisconsin card sorting test

The Wisconsin card sorting test (WCST) was used to assess the ability of abstract thinking. It consisted of 128 cards made up of different colors (red, yellow, green, and blue), shapes (triangle, cross, circle, and pentacle), and the number of shapes. The four templates were 1 red triangle, 2 pentacles, 3 yellow crosses, and 4 blue circles. In this research, the indexes used to evaluate cognitive function were: total test number, correct response number, wrong response number, persistent wrong response number, non-persistent wrong response number and completed classification number (35, 36).

#### Integrated visual and auditory continuous performance test

The Integrated visual and auditory continuous performance test (IVA-CPT) was used to evaluate the degree of dysfunctions in response control, attention and audiovisual integration in children. Persistent operation test software (US, Braintrain, IVA-CPT 3.0) was used to test the enrolled subjects. The IVA database automatically recorded the response control quotient, attention quotient and 29 other quotient indexes. During this study, the auditory control quotient, auditory attention quotient, visual control quotient, visual attention quotient, comprehensive control quotient and comprehensive attention quotient were selected to assess persistent attention deficits in children with ADHD (37–39).

### Genotyping

**FIGURE 2:**
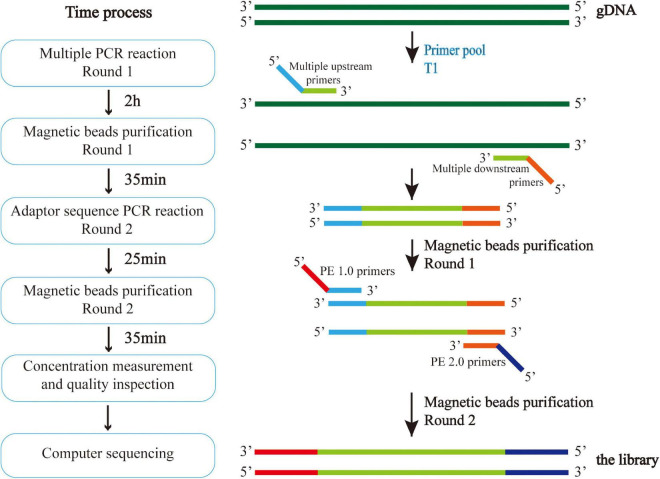
Multiple PCR technology was used to amplify multiple target regions of genomic DNA at the same time to obtain the amplicon. Then, the second generation sequencing connector was added to both sides of the amplicon by PCR to obtain the amplicon library, and the second generation sequencing was carried out to obtain the sequence information of the target region. The experiment was conducted in strict accordance with the library construction process.

DNA was extracted from venous blood by the Phenol-Chloroform method. Multiple polymerase chain reaction (PCR) targeted capture sequencing technology was used for gene analysis. For each coding sequence of the selected gene locus, a 120 bp probe sequence was designed from the first base in the direction of 5′–3′ according to the principle of sequence reverse complementation, and there was a 60 bp overlap between each two adjacent probe sequences. Sequences of *CAAGCAGAAGACGGCATACGAGAT* and *GTGACTGGAGTTCAGACGTG* were added to the 5′ and 3′ ends of each probe sequence, respectively; *in situ* synthesis of oligonucleotides was carried out on a large scale on a chip; the oligonucleotides on the chip were eluted with ammonia water and dissolved in water to form an oligonucleotide mixture. And through PCR, forward and reverse primers (Supplementary Table 1) with the 5′-end biotin-labeled oligonucleotide mixture were amplified to form a DNA probe library with biotin-labeled ADHD-related genes. The multi-PCR library construction and acquisition process is shown in Figure 2.

### Bioinformatics analysis

**FIGURE 3:**
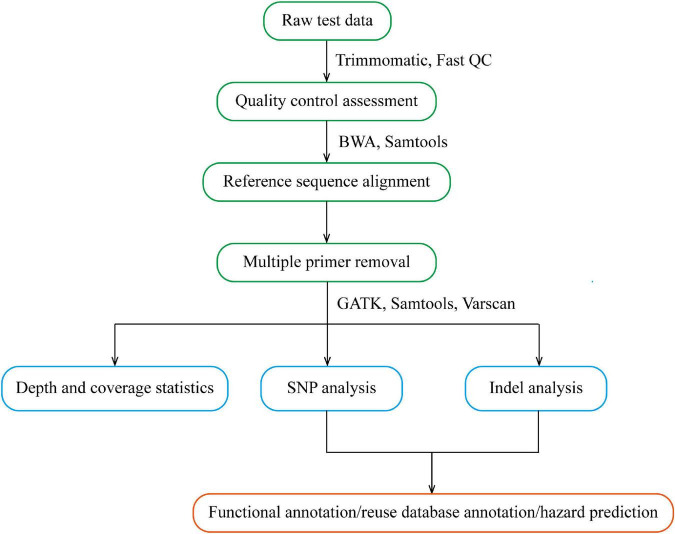
Flow chart of multiple PCR bioinformatics analysis.

After the original Sequenced Reads were obtained, the information analysis process was carried out by referring to the genome (GRCh38/HG38), including sequencing data quality assessment and variation detection. The former mainly carries out statistics on data volume, base quality, comparison rate, coverage rate, capture rate, uniformity and other indicators, and evaluates whether the database sequencing meets the standards. If it meets the standards, subsequent analyses will be conducted. The latter, compares high-quality sequences to the human reference genome, detects the variation information in the sample, and also makes statistics and annotations of the detected variation. The multiplex PCR bioinformatics analysis process is shown in Figure 3.

### Statistics

The double-check method was adopted for data entry. SPSS statistic (Version 24.0)^1^ was used to test the genetic balance fit degree of the Hardy–Weinberg law on population data. Correlation analysis was conducted between the indicators in the questionnaire results and the mutation of SNPs. R software (Version 4.0)^2^ was used to search for independent risk factors by multivariate logistic regression and the “corrplot” package was used for correlation analysis. A correlation was considered positive if the value of the correlation coefficient (rho) was >0, moderate positive if the rho was >0.2, weak negative if the rho was −0.2 ∼ 0, and moderate negative if the rho was ≤−0.2.

## Results

### Coincidence test of Hardy–Weinberg equilibrium

Sequencing and information analysis were performed on 23 loci of ADHD children, and no mutations were detected in 6 loci (rs11420276, rs12741964, rs11591402, rs28411770, rs11564750, and rs74760947). The coincidence test of Hardy–Weinberg equilibrium was performed on 17 loci with mutations. The results showed that the observations of 15 loci fit well with the expectations, conforming to Hardy–Weinberg equilibrium (*P* > 0.05), which was representative of the population, suggesting that the population in this study was genetically balanced. However, the genotype frequency distribution of rs1222063 and rs10044618 was not consistent with the Hardy–Weinberg equilibrium test (*P* < 0.05), and was therefore, not representative of the population. The results are shown in Table 1.

**TABLE 1:** Coincidence test of Hardy–Weinberg equilibrium of 17 attention deficit hyperactivity disorder (ADHD)-related loci in 193 children with ADHD.

Index variant	Genes	Genotypes	Observations (*n*)	Expectations (*n*)	Genotype frequency	Chi-square value	*P*-value
rs3768046	TIE1					4.438	0.109
		AA	4	2	0.021		
		AG	27	32	0.140		
		GG	162	160	0.839		
rs11210892	/					0.589	0.745
		GG	15	13	0.078		
		GA	70	74	0.363		
		AA	108	106	0.560		
rs2906457	ST3GAL3					1.623	0.444
		AA	31	27	0.161		
		AC	82	90	0.425		
		CC	80	76	0.415		
rs4858241	/					0.065	0.968
		TT	128	129	0.663		
		TG	59	58	0.306		
		GG	6	7	0.031		
rs429699	SLC6A3					7.663	0.022
		TT	6	13	0.031		
		TC	90	75	0.466		
		CC	97	104	0.503		
rs4916723	LINC00461					0.000	1.000
		AA	79	79	0.409		
		AC	89	89	0.461		
		CC	25	25	0.130		
rs1427829	/					0.158	0.924
		AA	29	30	0.150		
		AG	95	92	0.492		
		GG	69	70	0.358		
rs1410739	OBI1-AS1					0.895	0.639
		CC	10	8	0.052		
		CT	58	62	0.301		
		TT	125	123	0.648		
rs281324	SEMA6D					0.591	0.744
		TT	1	2	0.005		
		TC	37	35	0.192		
		CC	155	156	0.803		
rs212178	LINC01572					0.372	0.830
		GG	8	7	0.041		
		GA	56	59	0.290		
		AA	129	128	0.668		
rs1199039	TIE1					1.910	0.385
		AA	156	154	0.808		
		AG	33	37	0.171		
		GG	4	2	0.021		
rs1222063	/					13.642	0.001*
		GG	106	115	0.549		
		GA	86	68	0.446		
		AA	1	10	0.005		
rs9677504	SPAG16					2.512	0.285
		GG	121	124	0.627		
		GA	68	61	0.352		
		AA	4	7	0.021		
rs223508	MANBA					0.110	0.946
		CC	115	116	0.596		
		CT	69	67	0.358		
		TT	9	10	0.047		
rs27048	SLC6A3					0.131	0.937
		CC	151	152	0.782		
		CT	40	39	0.207		
		TT	2	3	0.010		
rs2652511	SLC6A3					2.907	0.234
		AA	144	141	0.746		
		AG	42	48	0.218		
		GG	7	4	0.036		
rs10044618	/					17.853	0.000*
		CC	166	161	0.860		
		CT	21	30	0.109		
		TT	6	1	0.031		

*P* > 0.05 means that the observations are in good agreement with the expectations, and conform to Hardy–Weinberg equilibrium; *P* < 0.05* means the opposite.

### Correlation analysis between single nucleotide polymorphisms and the severity of clinical symptoms of attention deficit hyperactivity disorder

**FIGURE 4:**
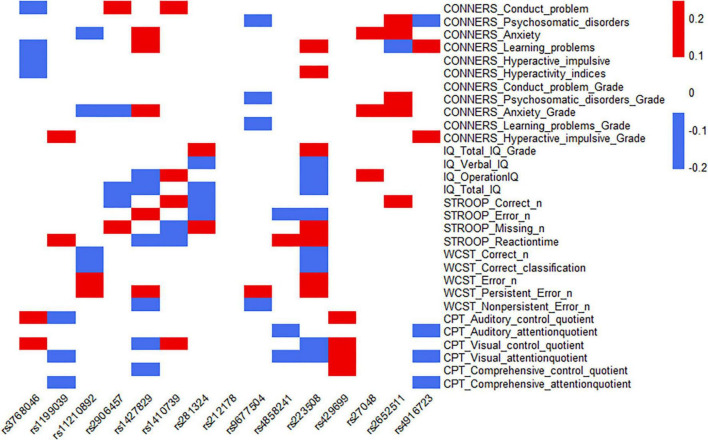
Correlation analysis between SNPs and the severity of clinical symptoms of ADHD.

The 15 SNPs obtained in the previous step and the clinical symptom characteristics of ADHD were analyzed, including 30 items from C-WISC, PSQ, Stroop color-word test, WCST and IVA-CPT; from this, the heatmap was made. The results showed that one SNP (rs9677504) and the psychosomatic disorders of PSQ had a moderate-negative correlation (the rho was −0.20), and a moderate-positive correlation with the persistent error number of WCST (the rho was 0.23); one SNP (rs223508) had a moderate-negative correlation with verbal IQ (the rho was −0.21), a moderate-negative correlation with correct numbers of WCST (the rho was −0.20), and a moderate-negative correlation with error numbers of WCST (the rho was −0.20); another SNP (rs2652511) had a moderate-positive correlation with anxiety (Grade) of PSQ (the rho was 0.24). Some SNPs, rs1427829 and rs223508, showed relatively high correlation coefficients with ADHD clinical symptoms (the rho >0.10); they had the largest number of items, 11 and 15, respectively. One SNP (rs212178) had a low correlation with the clinical symptoms and characteristics of ADHD, and there was no correlation coefficient of more than 0.10. The results are shown in Figure 4, and the specific correlation coefficients can be seen in Supplementary Table 2.

### Multivariate analysis results of the influence of single nucleotide polymorphisms on IQ differences of children with attention deficit hyperactivity disorder

As shown in Supplementary Table 3, multivariate analysis showed that one SNP (rs223508) was an independent risk factor for the decrease of VIQ (OR = 0.98, *P* = 0.006) and FIQ (OR = 0.98, *P* = 0.020) of ADHD children.

### Multivariate analysis results of the influence of single nucleotide polymorphisms on various clinical phenotypes of attention deficit hyperactivity disorder

Based on the results of the PSQ questionnaire, 5 SNPs have an impact on various clinical phenotypes of ADHD. Multivariate analysis showed that one SNP (rs1427829) was an independent risk factor for anxiety (OR = 4.89, *P* < 0.001) and anxiety (Grade) (*P* = 0.020), and similarly, one SNP (rs2652511) was an independent risk factor for anxiety (OR = 2.41, *P* = 0.024) and anxiety (Grade) (*P* = 0.003). It was also found that two SNPs were independent risk factors for conduct problem (rs2906457, OR = 2.08, *P* = 0.046; rs2906457, OR = 2.71, *P* = 0.044); one SNP (rs9677504) was an independent protective factor for psychosomatic disorders (OR = 0.27, *P* = 0.002). The results are shown in Table 2.

**TABLE 2:** Multivariate analysis results of the influence of single nucleotide polymorphisms (SNPs) on various clinical phenotypes of ADHD.

PSQ itmes	rs1427829		rs9677504		rs2652511		rs2906457		rs1410739	
	OR [95% CI]	*P*-value	OR [95% CI]	*P*-value	OR [95% CI]	*P*-value	OR [95% CI]	*P*-value	OR [95% CI]	*P*-value
Conduct problems	1.00 [0.47, 2.11]	0.992	1.12 [0.65, 1.93]	0.694	1.09 [0.59, 2.02]	0.777	2.08 [0.97, 4.48]	0.046*	2.71 [0.78, 9.49]	0.044*
Conduct problems (Grade)	/	0.693	/	1	/	0.734	/	1	/	0.556
Psychosomatic disorders	0.55 [0.22, 1.41]	0.371	0.27 [0.11, 0.67]	0.002*	1.85 [0.82, 4.16]	0.143	1.50 [0.53, 4.25]	0.371	3.43 [0.47, 25.1]	0.115
Psychosomatic disorders (Grade)	/	1	/	0.095	/	0.069	/	0.874	/	0.766
Anxiety	4.89 [1.43, 16.7]	<0.001*	1.25 [0.66, 2.33]	0.506	2.41 [1.20, 4.81]	0.024*	0.96 [0.43, 2.14]	0.910	1.26 [0.33, 4.77]	0.740
Anxiety (Grade)	/	0.020*	/	0.97	/	0.003*	/	0.256	/	0.928
Learning problems	1.61 [0.83, 3.14]	0.124	0.92 [0.57, 1.49]	0.748	0.73 [0.42, 1.26]	0.294	1.34 [0.73, 2.49]	0.290	0.72 [0.28, 1.86]	0.422
Hyperactive/impulsive	0.94 [0.51, 1.73]	0.833	1.25 [0.80, 1.95]	0.330	1.03 [0.62, 1.71]	0.888	1.19 [0.67, 2.12]	0.556	0.81 [0.34, 1.93]	0.629
Hyperactivity indices	1.25 [0.56, 2.77]	0.494	1.25 [0.71, 2.21]	0.445	0.89 [0.46, 1.70]	0.689	1.37 [0.65, 2.91]	0.384	1.10 [0.35, 3.46]	0.849

*Statistically significant.

### Multivariate analysis results of the influence of single nucleotide polymorphisms on control capabilities of children with attention deficit hyperactivity disorder

Combined with the results of the Stroop color-word test and multivariate analysis, as shown in Supplementary Table 4, one SNP (rs1410739) was found to be an independent risk factor affecting the control ability of ADHD children, which was embodied in the correct number of test items (OR = 1.03, *P* = 0.019). The SNP had no effect on the reaction time of test items (OR = 1.00, *P* = 0.040).

### Multivariate analysis results of the influence of single nucleotide polymorphisms on the ability for abstract thinking of children with attention deficit hyperactivity disorder

According to the test results of WCST, 6 SNPs had an impact on the abstract thinking ability of children with ADHD. The results of multivariate analysis showed that subjects with rs9677504 (OR = 0.95, *P* = 0.035) or rs3768046 (OR = 0.96, *P* = 0.030) mutations were less likely to have an increase in the non-persistent error number, and subjects with rs1427829 mutation had an increased likelihood of persistent errors (OR = 1.05, *P* = 0.038). One SNP (rs1410739) was found to be associated with the correct classification results (*P* = 0.049). There were two SNPs, rs11210892 and rs223508, that were found to share some similar and significant results; with those two mutations, children with ADHD were more likely to have an increase in the error number and persistent error number, and less likely to have an increase in the correct number, as shown in Table 3. It can be inferred that rs9677504, rs1427829, rs1427829 and rs1427829 are independent risk factors for ADHD symptom aggravation, and rs3768046 is an independent protective factor.

**TABLE 3:** Multivariate analysis results of the influence of SNPs on the ability for abstract thinking of children with ADHD.

WCST	rs9677504		rs1427829		rs11210892		rs223508		rs1410739		rs3768046	
	OR [95% CI]	*P*-value	OR [95% CI]	*P*-value	OR [95% CI]	*P*-value	OR [95% CI]	*P*-value	OR [95% CI]	*P*-value	OR [95% CI]	*P*-value
Correct number	0.98 [0.95, 1.02]	0.332	0.99 [0.94, 1.04]	0.659	0.92 [0.85, 1.00]	0.036*	0.95 [0.91, 0.98]	0.005*	0.99 [0.92, 1.06]	0.709	0.95 [0.83, 1.09]	0.354
Correct classification	/	0.411	/	0.476	/	0.172	/	0.323	/	0.049*	/	0.654
Error number	1.02 [0.98, 1.06]	0.332	1.01 [0.96, 1.06]	0.659	1.09 [1.00, 1.18]	0.036*	1.05 [1.02, 1.09]	0.005*	1.01 [0.94, 1.09]	0.709	1.05 [0.92, 1.20]	0.354
Persistent error number	1.05 [1.01, 1.09]	0.059	1.05 [0.99, 1.12]	0.038*	1.08 [0.98, 1.20]	0.003*	1.04 [1.01, 1.08]	0.016*	0.98 [0.92, 1.05]	0.581	1.12 [0.91, 1.37]	0.125
Non-persistent error number	0.95 [0.90, 1.00]	0.035*	0.95 [0.89, 1.01]	0.053	1.03 [0.93, 1.13]	0.578	1.00 [0.95, 1.05]	0.923	1.07 [0.96, 1.19]	0.226	0.96 [0.83, 1.11]	0.030*

*Statistically significant.

### Multivariate analysis results of the influence of single nucleotide polymorphisms on persistent attention deficits of children with attention deficit hyperactivity disorder

The IVA-CPT is a tool to reflect the degree of children’s response control, attention and audiovisual integration dysfunction. According to the test results, we conducted a multivariate analysis, and as shown in Supplementary Table 5, the results showed that one SNP (rs4916723) was an independent risk factor for the decrease of visual attention quotient (OR = 0.99, *P* = 0.036) in ADHD children.

## Discussion

Previous studies have confirmed that the occurrence of ADHD is affected by multiple genes. In this research, various ADHD-related questionnaires were used to reflect the severity of the disease, and the correlation between the single nucleotide polymorphism of specific sites and the severity of clinical symptoms was then analyzed. The results presented that the SNPs at different loci have an impact on the severity of clinical symptoms of ADHD, a supplement to the field of genetic research on ADHD. This indicates a role for genetic testing in the evaluation of children with ADHD. Additionally, when the same SNP shows statistical differences in several questionnaires or several sub-items of the same questionnaire, the evaluation significance of the odds ratio of different questionnaires and items is consistent, which confirms the reliability of our study.

Based on the strong evidence that the dopaminergic neurotransmission system is involved in ADHD, the gene encoding dopamine transporter (DAT) on human chromosome 5 (SLC6A3 gene; also known as DAT1) has been proposed as a candidate gene for ADHD (25, 26). SLC6A3 may alter human DA transporter protein (hDAT) density, DA reuptake activity, and the dynamics of DA neurotransmission, participating in the pathophysiology of the central and peripheral nervous systems (40). Studies on the genetic basis of individual differences in attention suggest that SLC6A3 polymorphism is associated with executive attention (41, 42). Recent studies have also shown that the SLC6A3 genotype affects attention/cognitive function (26). Our results showed that rs2652511 (SLC6A3 locus) was an independent risk factor for the anxiety symptom in children with ADHD, which is new information on SLC6A3 gene research. It should be noted that there was no significant difference in this SNP on the questionnaire reflecting attention, which may be caused by the fact that our study was conducted on the symptoms of children with ADHD, or may be caused by probable reasons that the SLC6A3 gene maybe works in the form of multi-gene combination and it requires further study in the future.

Among the 9 loci with significantly different results in multivariate analysis, rs1410739 (OBI1-AS1 locus) affected three items from the questionnaire, including PSQ, STROOP, and WCST, respectively, corresponding to conduct problems, control ability and abstract thinking ability of children with ADHD, and all were independent risk factors. This may indicate a meaningful SNP locus that can simultaneously affect multiple ADHD symptoms was identified. Further it would provide a basis for future studies on ADHD and SNP. The SNP rs1410739 (locus in OBI-AS1, which is also regarded as RNF219-AS1) was found to be significantly associated with ADHD in the recent TWAS based on ADHD GWAS (21), however, the mechanism has not been effectively explained, so it is reasonable to discuss results at the level of the OBI1-AS1 gene. Although the exact function of OBI1-AS1 remains unclear, several studies have suggested a regulatory role for the hybridization of natural antisense RNAs with endogenous mRNAs (43). Recent studies have reported that RNF219-AS1 was involved in the pathophysiology of ADHD (44), and that OBI1-AS1 has a potential role in glutamate receptor signaling and synaptic responses (45). Previous studies have linked genetic variations in the ion glutamate receptor to ADHD risk (46). Several human genetic studies and animal studies also reported that genetic variations in the metabotropic glutamate receptor (mGluRs) subtype III may be associated with ADHD (47–54). OBI1-AS1 expression has also been reported to be associated with astrocyte content, and some studies in recent years have attributed the pathophysiology of depression and mood disorders to astrocytes (45, 55–57). These can aid in the interpretation of our results to some extent, but our findings must be viewed with caution and be seen as preliminary. This research will need to be supported by further research.

In our study, rs3768046 (TIE1 locus) was found to influence the abstract thinking ability of children with ADHD. The latest study reported that rs3768046 may change the expression of TIE1 by affecting the binding sites of transcription factors, and the expression level of TIE1 in the blood samples of patients was significantly higher than that of the control group, suggesting that TIE1 is a susceptibility gene for ADHD. The study also indicated that the G allele of ra3768046 was associated with increased susceptibility to ADHD in Chinese Han children (58). Previous studies have indicated that TIE1 plays a key role in normal vascular development and function by forming a heteromeric complex with another TIE receptor, TIE2 (59–62). The overexpression of TIE1 may lead to the destruction of the TIE2 signaling system, thus affecting the normal development and function of the vascular system, leading to neuroinflammation and the destruction of the blood–brain barrier, etc., and then damaging ADHD-related organs and systems. Abnormal perfusion may be one of the pathological bases of TIE1’s effect on ADHD (58, 63, 64).

Another SNP, rs223508 (MANBA locus), was found to be an independent risk factor to influence VIQ, IQ and abstract thinking ability via multivariate analysis. The protein β-mannosidase encoded by MANBA is a member of the glycosylhydrolase 2 family and acts as the final exon of β-glycosidase in the N-linked glycoprotein oligosaccharide catabolism pathway (65). MANBA is mainly confined to the midbrain and hindbrain, including the cerebellar cortex, medulla and pons. The expression level of MANBA is very low at all stages of human brain development (66), and its activity decreases with age (67). Up to date, there are few studies on MANBA and ADHD, and the mechanism is not clear. Recent studies have found that the gene-regulated expression of MANBA in the cerebellum is significantly associated with the risk of ADHD (68), and the expression of MANBA is significantly up-regulated in patients (58). In contrast, other studies have shown that increased levels of β-mannosidase are associated with a reduced risk of ADHD (69). More research is needed to resolve these controversies.

We also found that rs2906457 (ST3GAL3 locus) had an effect on the severity of conduct problems in children with ADHD, and that it was its independent risk factor. ST3GAL3 is expressed in a variety of tissues, including neurons, and encodes a membrane protein (ST3Gal III) that adds sialic acid to the end of glycolipids or glycoproteins, a process that has an important impact on brain function, and St3gal3 single gene deletion mice have reduced motor coordination, cognitive impairment and behavioral hyperactivity (70, 71). Haploid deficiency of ST3GAL3 leads to sex-dependent changes in markers of cognitive, behavioral, and brain plasticity (72). Since the human brain is particularly rich in sialic acid containing glycolipids (gangliosides), ST3GAL3 may also play a role in human brain development (73). It has been indicated that gangliosides regulate calcium homeostasis and signal transduction in neurons (74). Recent human gene analysis has indicated that increased transcription of ST3GAL3 is significantly associated with ADHD, and common gene variants in ST3GAL3 are also associated with education level (75–77). Our results further confirmed the functional correlation between ST3GAL3 and ADHD.

The SNP rs4916723 (LINC00461 locus) was found to affect response control, attention, and audiovisual integration. This SNP is located in the gene LINC00461, which is mainly expressed in the brain and plays a key role in the regulation of brain function (78–80). LINC00461 is one of the most pleiotropic genome-wide risk genes for major psychiatric traits, and it has been found to be associated with five psychiatric disorders at the same time, including ADHD, depression, anxiety disorder, schizophrenia and the personality trait of neuroticism (81–83). The latest study also reports LINC00461 as a novel risk gene for ASD and confirms that the LINC00462-MEF2C gene cluster is one of the most potent genomic contributors to major psychotic traits (84). Moreover, a recent GWAS study said that LINC00461 correlates with education and is associated with reduced brain size, cortical morphology abnormalities, and hippocampal mossy fiber morphology abnormalities (76). Knockout of LINC00461 lineal homolog expression in mouse embryos showed impaired neuronal migration, further supporting its role in the neurodevelopmental hypothesis of most major psychiatric disorders (81). Previous studies also indicated that some lncRNAs in the cytoplasm may bind to miRNAs to inhibit their activity, thereby regulating the expression of target genes (85). It is worth mentioning that MIR9-2, which encodes neuron-specific miRNA miR-9, is located within the loci LINC00461. Knockdown of LINC00461 also greatly inhibits the expression level of Mir-9 (78, 86), while overexpression of miR-9 has been shown to be sufficient to ameliorate neural migration defects (87).

What’s more, we found that rs1427829 (intron) could influence the severity of anxiety symptoms and abstract thinking ability in children with ADHD, also, rs11210892 (intron) had an effect on abstract thinking ability in children with ADHD. They have previously been found to be significantly associated with the development of ADHD in multiple replication mete-analyses (7, 21). Besides, we found that rs9677504 (SPAG16 locus) affects the severity of psychosomatic disorders and abstract thinking ability. Previous studies have shown that SPAG16 is associated with osteoporosis (88), primary ciliary motility disorder (PCD) (89), multiple sclerosis (90, 91), and reduced male fertility due to arsenism and fluorosis (92, 93). However, the mechanism of these three SNPs, with respect to their impact on the onset and severity of ADHD symptoms, has not been explained yet.

In addition, the use of “with or without” grading of PSQ projects according to the literature was verified to a certain extent. Table 2 shows that “Anxiety (Grade)” and “Anxiety,” which have significant results in children with ADHD, are always consistent. When accounting for possible genetic differences between populations, our study is also a validation of these SNPs in a sample of Chinese children.

There are several limitations of our work that should be considered. First, our sample size is relatively small, so it needs to be expanded in the future in order to provide more reliable results. Second, our susceptibility SNPs were screened according to the results of previous research including GWAS, TWAS and et al., which may cause that not all ADHD-related SNPs were included in our study. Third, we focused only on children with ADHD and did not perform a comparative analysis with healthy controls. Fourth, although our research has achieved certain results, the underlying mechanism of SNPs is still unclear, which requires further cell and animal experiments to explore and verify. In the future, we plan to conduct further studies on the clinical phenotypes and neural mechanisms of ADHD in combination with more clinical data from these children.

## Data availability statement

The original contributions presented in this study are publicly available. This data can be found here: NCBI: PRJNA873257.

## Ethics statement

The studies involving human participants were reviewed and approved by the Ethics Committee of Clinical Research in The First Affiliated Hospital of Wenzhou Medical University. Written informed consent to participate in this study was provided by the participants’ legal guardian/next of kin.

## Author contributions

YX and MW: conceptualization. YX, JT, AQ, XL, RZ, SC, CY, and BC: acquisition of the data for the work. YX and SL: analysis of data for the work. YX, SL, and AQ: interpretation of data for the work. YX, SL, and PV: manuscript writing. MW and AQ: funding acquisition. All authors contributed to the article and approved the submitted version.

## Footnotes

1
https://www.ibm.com/products/spss-statistics

2
https://www.r-project.org/
