# Supplementary material for: Correlation research of susceptibility single nucleotide polymorphisms and the severity of clinical symptoms in attention deficit hyperactivity disorder
Source: Front Psychiatry. 2022 Sep 23;13:1003542. doi: 10.3389/fpsyt.2022.1003542 (PMC9538111; doi:10.3389/fpsyt.2022.1003542)
Supplement: Supplementary file 1 [file Data_Sheet_1.docx]

**Supplementary Table 1. Forward and reverse primers in genotyping procedure.**

| **Index variant** | **Forward Primer** | **Reverse Primer** |
| --- | --- | --- |
| rs3768046 | TTGTGCCAGCAAGACTGAGTAC | ACTCATTCTGCCTTTGAGCCTC |
| rs1199039 | TACCCACGAAGACTGACTCCTT | AAGGGTGAGGGTTGAATGAGAC |
| rs11210892 | TGCTGGGTGGTTATGTTCCCTA | CAACACAGCTGGATGTGAGACT |
| rs11420276 | TCGATCTCCCGATCTCATGATCC | AAGGGAAAAACACATTAGGAAGGCT |
| rs12741964 | GCACAGTGATGGCCTAAAGAAG | CCAAGAGATGCATGCCTTCATG |
| rs2906457 | TGGGAAAGACCTTCATGGGAAG | TCTGCTCCAAATCCTCCATTCC |
| rs1222063 | CTGAGGCAGGAGAATTGTTTGAAC | CCAAGTATTCTCATTGCTTTCTGGC |
| rs11591402 | GCAGCCTGAGATTTCTCTTTTCTTC | TGGTGGGTGCGTTCTGAATAAT |
| rs1427829 | ATTTGAACCCCAGGAGAGTTGC | AGCATCTTCCTATGGAGGGGTT |
| rs1410739 | CAGGCTAGGCTAAAGTACTTCCAA | TCAGTTTGCCTTTTGCTTATGACC |
| rs281324 | AACCGACAGATAGTCCCAAAGG | CATGTGGCAGGATCAAACCTTG |
| rs212178 | TCGAGTTCTGCTAAGGGACAGA | ACTCTAGACCCTTTTTGCGTGG |
| rs9677504 | GCTTGGGCTGATAATGCAACAC | TCTCTGTGTGACCTTGGTCAGA |
| rs4858241 | CCCTCTTAGTACTGCTTGCTGT | CAAGCATCTTCTCTGATCACAAGG |
| rs28411770 | ACGTCATGAGAAAGATGCATGAGA | GTCTGGGATATGGTCATGCACTT |
| rs223508 | CTTCCACAGCAACATGGATGGA | TGCTTGTACCCATTCATCAGCC |
| rs429699 | ATCCAAGCTAAGCACCTCACTG | ATCCTCTTTGGAGTGCTCATCG |
| rs27048 | TACCTTGCTATCCCTGCAAGC | GCAAGGCTTCTGAACTGGTCA |
| rs2652511 | TCGCACTCGCCTAAGAAAACC | ACTCACCTCGGTGCCTTCTAA |
| rs11564750 | ACCCAAAGCTAACAGGGTACAG | GGAATGCTCTTTGTCTTGGCAG |
| rs10044618 | GAACATGCATGCACTGATGACAG | CAGAGGGGACCAAACATAAACCA |
| rs4916723 | TGAAGCTCCTCAGTCTACCCAT | AGACCGGATGCATAATGAGCC |
| rs74760947 | TGAAGCTCCTCAGTCTACCCAT | AGACCGGATGCATAATGAGCC |

**Supplementary Table 2. Correlation coefficient between SNPs and the severity of clinical symptoms of ADHD.**

| **Items** | **rs3768046** | **rs1199039** | **rs11210892** | **rs2906457** | **rs1427829** | **rs1410739** | **rs281324** | **rs212178** | **rs9677504** | **rs4858241** | **rs223508** | **rs429699** | **rs27048** | **rs2652511** | **rs4916723** |
| --- | --- | --- | --- | --- | --- | --- | --- | --- | --- | --- | --- | --- | --- | --- | --- |
| PSQ |  |  |  |  |  |  |  |  |  |  |  |  |  |  |  |
| Conduct problems | -0.14* | 0.03 | 0.07 | 0.11* | 0.00 | 0.11* | -0.08 | -0.04 | 0.02 | 0.01 | 0.05 | 0.02 | 0.03 | 0.02 | 0.04 |
| Psychosomatic disorders | -0.06 | -0.06 | -0.07 | 0.00 | -0.10 | 0.08 | -0.05 | -0.10 | -0.20* | -0.05 | 0.08 | 0.05 | 0.00 | 0.13* | -0.13* |
| Anxiety | 0.03 | 0.08 | -0.14* | -0.03 | 0.19* | 0.02 | 0.00 | -0.01 | 0.05 | -0.01 | -0.01 | -0.09 | 0.12* | 0.19* | 0.08 |
| Learning problems | -0.10* | 0.01 | -0.09 | 0.06 | 0.10* | -0.04 | 0.06 | 0.00 | -0.04 | 0.04 | 0.12* | 0.08 | -0.07 | -0.11* | 0.11* |
| Hyperactive impulsive | -0.11* | 0.01 | -0.02 | 0.04 | -0.01 | -0.09 | -0.06 | -0.06 | 0.06 | 0.07 | 0.04 | 0.04 | 0.05 | 0.01 | -0.06 |
| Hyperactivity indices | -0.11* | 0.01 | -0.05 | 0.05 | 0.04 | -0.02 | -0.03 | -0.05 | 0.04 | 0.00 | 0.12* | 0.06 | 0.01 | -0.04 | 0.02 |
| Conduct problems  (Grade) | -0.04 | 0.06 | 0.02 | -0.04 | 0.02 | -0.06 | -0.02 | -0.06 | -0.03 | 0.08 | -0.03 | -0.04 | 0.08 | 0.04 | 0.03 |
| Psychosomatic disorders (Grade) | -0.03 | -0.05 | -0.08 | -0.06 | 0.00 | 0.05 | -0.09 | -0.08 | -0.11* | -0.07 | 0.10 | 0.07 | 0.00 | 0.18* | -0.07 |
| Anxiety (Grade) | 0.06 | 0.03 | -0.17* | -0.12* | 0.18* | 0.08 | -0.08 | 0.01 | 0.02 | 0.04 | 0.07 | -0.05 | 0.10* | 0.24* | 0.05 |
| Learning problems (Grade) | -0.02 | -0.03 | -0.04 | -0.06 | 0.04 | -0.04 | -0.01 | -0.03 | -0.11* | 0.03 | 0.05 | -0.03 | -0.01 | 0.00 | 0.03 |
| Hyperactive impulsive (Grade) | -0.05 | 0.11* | -0.02 | -0.05 | 0.05 | -0.08 | -0.03 | 0.01 | 0.01 | 0.09 | 0.02 | -0.06 | 0.01 | 0.07 | 0.12* |
| IQ |  |  |  |  |  |  |  |  |  |  |  |  |  |  |  |
| Verbal IQ | 0.01 | 0.04 | 0.03 | -0.04 | -0.07 | 0.01 | -0.14* | 0.01 | -0.01 | -0.02 | -0.21* | -0.05 | 0.02 | 0.04 | -0.04 |
| Operation IQ | -0.01 | 0.04 | 0.09 | -0.06 | -0.13* | 0.12* | -0.08 | 0.09 | 0.01 | -0.04 | -0.13* | -0.01 | 0.12* | 0.08 | -0.06 |
| Total IQ | 0.01 | 0.09 | -0.01 | -0.10* | -0.11* | 0.07 | -0.13* | 0.07 | -0.03 | -0.08 | -0.17* | -0.03 | 0.05 | 0.03 | -0.07 |
| Total IQ (Grade) | 0.01 | -0.08 | 0.00 | 0.10 | 0.08 | -0.07 | 0.11* | -0.09 | 0.05 | 0.05 | 0.17* | 0.03 | -0.02 | -0.04 | 0.07 |
| STROOP |  |  |  |  |  |  |  |  |  |  |  |  |  |  |  |
| Correct number | 0.07 | 0.05 | -0.03 | -0.10* | 0.00 | 0.11* | -0.13* | 0.00 | 0.02 | 0.02 | -0.08 | 0.03 | 0.08 | 0.10* | 0.02 |
| Error number | 0.00 | -0.05 | 0.03 | -0.06 | 0.13* | 0.04 | -0.14* | 0.02 | 0.07 | -0.12* | -0.11* | 0.01 | 0.00 | -0.03 | -0.01 |
| Missing number | -0.07 | -0.02 | 0.01 | 0.11* | -0.08 | -0.11* | 0.12* | -0.03 | -0.06 | 0.05 | 0.12* | -0.03 | -0.08 | -0.07 | -0.02 |
| Reaction time | -0.06 | 0.15* | -0.01 | 0.03 | -0.11* | -0.12* | 0.06 | -0.02 | -0.03 | 0.16* | 0.12* | -0.08 | -0.03 | 0.06 | -0.04 |
| WCST |  |  |  |  |  |  |  |  |  |  |  |  |  |  |  |
| Correct number | -0.05 | -0.02 | -0.15* | 0.02 | -0.03 | 0.00 | -0.03 | -0.06 | -0.09 | -0.04 | -0.20* | 0.05 | 0.02 | -0.02 | 0.05 |
| Correct classification | 0.01 | -0.05 | -0.11* | 0.02 | -0.04 | -0.01 | -0.01 | -0.01 | 0.01 | -0.07 | -0.15* | 0.09 | 0.09 | 0.07 | 0.00 |
| Error number | 0.05 | 0.02 | 0.15* | -0.02 | 0.03 | 0.00 | 0.03 | 0.06 | 0.09 | 0.04 | 0.20* | -0.05 | -0.02 | 0.02 | -0.05 |
| Persistent error number | 0.08 | -0.05 | 0.11* | -0.04 | 0.12* | -0.06 | 0.01 | 0.06 | 0.23* | -0.02 | 0.16* | -0.08 | 0.05 | 0.04 | 0.02 |
| Non-persistent error number | -0.04 | 0.09 | 0.04 | 0.03 | -0.13* | 0.09 | 0.03 | 0.00 | -0.17* | 0.09 | 0.01 | 0.05 | -0.10 | -0.03 | -0.10 |
| CPT |  |  |  |  |  |  |  |  |  |  |  |  |  |  |  |
| Auditory control quotient | 0.11* | -0.11* | 0.03 | -0.06 | -0.03 | 0.05 | 0.02 | 0.00 | -0.04 | -0.04 | -0.01 | 0.12* | -0.05 | 0.02 | -0.07 |
| Auditory attention quotient | -0.01 | -0.05 | 0.01 | -0.02 | 0.01 | 0.05 | -0.05 | 0.03 | 0.05 | -0.10* | -0.04 | 0.04 | 0.07 | 0.04 | -0.14* |
| Visual control quotient | 0.10* | -0.04 | 0.01 | -0.04 | -0.11* | 0.11* | -0.07 | -0.04 | -0.07 | 0.07 | -0.10* | 0.12* | -0.01 | 0.01 | -0.01 |
| Visual attention quotient | 0.09 | -0.15* | 0.03 | -0.01 | -0.09 | 0.03 | -0.05 | 0.00 | 0.03 | -0.14* | -0.11* | 0.11* | 0.08 | -0.03 | -0.15* |
| Comprehensive control quotient | 0.08 | -0.06 | -0.02 | -0.06 | -0.10* | 0.08 | -0.02 | -0.02 | -0.08 | 0.01 | -0.09 | 0.16* | -0.02 | 0.02 | -0.07 |
| Comprehensive attention quotient | 0.08 | -0.13* | 0.01 | 0.00 | -0.03 | 0.05 | -0.06 | 0.05 | 0.05 | -0.10 | -0.08 | 0.08 | 0.10 | 0.00 | -0.13* |

**Supplementary Table 3. Multivariate analysis results of the influence of SNPs on IQ differences of children with ADHD.**

| **IQ itmes** | **rs223508** | |
| --- | --- | --- |
|  | **OR**  **[95%CI]** | **P value** |
| VIQ | 0.98 [0.96;0.99] | 0.006* |
| PIQ | 0.98 [0.97;1.00] | 0.102 |
| FIQ | 0.98 [0.96;1.00] | 0.020* |

**Supplementary Table 4. Multivariate analysis results of the influence of SNPs on control capabilities of children with ADHD.**

| **STROOP items** | **rs1410739** | |
| --- | --- | --- |
|  | **OR**  **[95%CI]** | **P value** |
| Correct number | 1.03 [0.99;1.07] | 0.019* |
| Error number | 1.00 [0.96;1.05] | 0.908 |
| Missing number | 0.98 [0.96;1.01] | 0.204 |
| Reaction time | 1.00 [1.00;1.00] | 0.040* |

**Supplementary Table 5. Multivariate analysis results of the influence of SNPs on persistent attention deficits of children with ADHD.**

| **CPT items** | **rs4916723** | |
| --- | --- | --- |
|  | **OR**  **[95%CI]** | **P value** |
| Auditory control quotient | 0.99 [0.98;1.01] | 0.362 |
| Auditory attention quotient | 0.99 [0.98;1.00] | 0.055 |
| Visual control quotient | 1.00 [0.99;1.01] | 0.829 |
| Visual attention quotient | 0.99 [0.98;1.00] | 0.036* |
| Comprehensive control quotient | 0.99 [0.98;1.01] | 0.358 |
| Comprehensive attention quotient | 0.99 [0.98;1.00] | 0.066 |
